# Imaging Liver Lesions Using Grating-Based Phase-Contrast Computed Tomography with Bi-Lateral Filter Post-Processing

**DOI:** 10.1371/journal.pone.0083369

**Published:** 2014-01-17

**Authors:** Julia Herzen, Marian S. Willner, Alexander A. Fingerle, Peter B. Noël, Thomas Köhler, Enken Drecoll, Ernst J. Rummeny, Franz Pfeiffer

**Affiliations:** 1 Institute of Materials Science, Helmholtz-Zentrum Geesthacht, Geesthacht, Germany; 2 Physics Department & Institute of Medical Engineering, Technische Universität München, Garching, Germany; 3 Department of Radiology, Technische Universität München, Munich, Germany; 4 Philips Technologie GmbH, Innovative Technologies, Research Laboratories, Hamburg, Germany; 5 Institute of Pathology, Technische Universität München, Munich, Germany; Kaohsiung Medical University Hospital, Kaohsiung Medical University, Taiwan

## Abstract

X-ray phase-contrast imaging shows improved soft-tissue contrast compared to standard absorption-based X-ray imaging. Especially the grating-based method seems to be one promising candidate for clinical implementation due to its extendibility to standard laboratory X-ray sources. Therefore the purpose of our study was to evaluate the potential of grating-based phase-contrast computed tomography in combination with a novel bi-lateral denoising method for imaging of focal liver lesions in an ex vivo feasibility study. Our study shows that grating-based phase-contrast CT (PCCT) significantly increases the soft-tissue contrast in the ex vivo liver specimens. Combining the information of both signals – absorption and phase-contrast – the bi-lateral filtering leads to an improvement of lesion detectability and higher contrast-to-noise ratios. The normal and the pathological tissue can be clearly delineated and even internal structures of the pathological tissue can be visualized, being invisible in the absorption-based CT alone. Histopathology confirmed the presence of the corresponding findings in the analyzed tissue. The results give strong evidence for a sufficiently high contrast for different liver lesions using non-contrast-enhanced PCCT. Thus, ex vivo imaging of liver lesions is possible with a polychromatic X-ray source and at a spatial resolution of ∼100 µm. The post-processing with the novel bi-lateral denoising method improves the image quality by combining the information from the absorption and the phase-contrast images.

## Introduction

Focal liver lesions are a common finding in ultrasound and computed tomography (CT) examinations often presenting without clinical symptoms [1]–[3]. Whether a hepatic lesion requires treatment, follow-up imaging, or does not need further attention depends on the dignity (benign vs. malignant) and entity of the finding [4]. Furthermore, in oncology the liver is the most common site of metastasis from gastrointestinal tumors. Accurate assessment of tumor spread and treatment response are important for therapy planning [5].

Non-enhanced CT is very limited for the detection and characterization of focal hepatic lesions due to its low soft-tissue contrast. The use of iodine contrast agents greatly improves detectability of lesions; however, the characterization especially of subcentimetric findings remains challenging [6]. Also, in some patients iodine-based contrast media can cause anaphylactic reactions, acute renal failure, or hyperthyroidism [7]–[9]. Drug interactions with metformin, an oral antidiabetic agent, have been described [10]. The gold standard for the detection and characterization of focal hepatic lesions remains magnetic resonance imaging (MRI) with the application of diffusion weighted imaging (DWI) and liver-specific contrast agents [11], [12]. Contraindications for MRI include cardiac pacemakers and patients suffering from claustrophobia. Additionally, long scanning times can be a limitation due to motion artifacts or limited patient tolerance, as well as the limited spatial resolution compared to CT.

Conventional multi-slice CT uses the attenuation of x-rays by photo-electric absorption and Compton scattering processes in the examined object as the sole source of contrast. Therefore, structures containing elements with high Z number, like calcium in bones, give high contrast whereas soft tissues such as muscle, parenchymatous organs and adipose tissue show rather low contrast. On the other hand, phase-contrast computed tomography (PCCT) exploits the physical effect of the phase shift x-rays experience when passing through an object.

Different phase-contrast methods have been developed over the last decades [13]. All these methods can be divided into three main groups: (1.) the direct methods measuring the phase shift (Bonse-Hart interferometer) [14], [15], (2.) the propagation based methods measuring the 2nd derivative of the phase shift [16], and (3.) the differential methods measuring the 1st derivative of the phase shift by using e.g. the analyzer-based [17], [18] or the grating-based method [19], [20]. Only the grating-based phase-contrast computed tomography (PCCT) has been successfully expanded to standard, polychromatic X-ray tubes [21]. This method provides a three-dimensional map of the attenuation coefficients and the electron density distribution of the specimen at the same time. Several recent articles have shown the potential of the grating-based PCCT for clinical applications demonstrating a remarkable improvement of soft-tissue contrast without the use of contrast media [22]–[25].

The purpose of our experimental proof-of-principle study was to evaluate the potential of grating-based PCCT for the detection and characterization of focal lesions in ex-vivo human liver specimens using a standard laboratory x-ray tube setup. Furthermore, we aimed to apply a novel post-processing denoising method on the tomographic data to demonstrate the possible quality improvement of the images.

## Materials and Methods

### Grating interferometry

**Figure 1:**
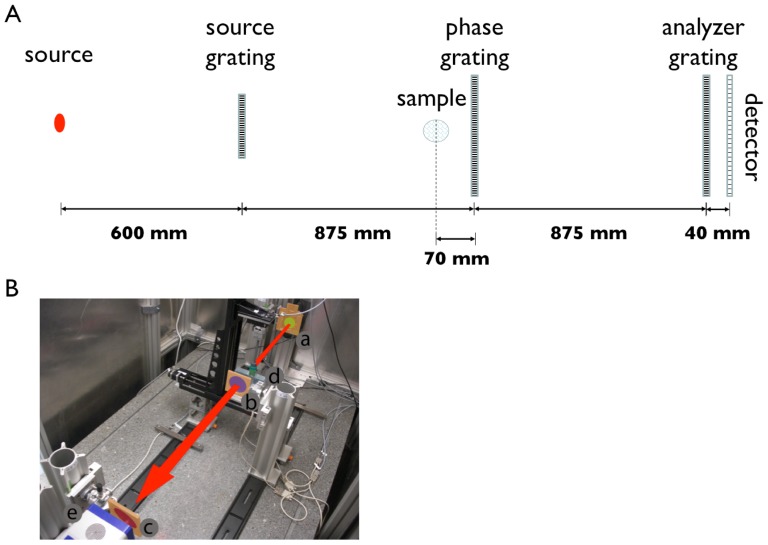
Schematic and a photograph of the grating-based imaging setup. A) Schematic top view of the grating-based imaging setup. The X-ray tube is a rotating anode with a Molybdenum target working with max. 60 kVp and 80 mA. The grating-interferometer consists of a source grating (to increase the coherence), a phase grating, an analyzer grating and a detector. The specimen is rotated in this setup. B) Photograph of the grating-based PCCT setup using conventional X-ray sources consisting of three gratings arranged in a symmetrical setup: (a) source grating marked yellow, (b) phase grating marked purple and (c) analyzer grating marked red, (d) the specimen mounted to a hanging rotation stage and (e) the detector. A red arrow marks the X-ray beam path.

The principle of grating-based PCCT and its projection acquisition is explained in detail in [19]–[21]. The x-rays are attenuated and refracted when they pass through the object. The refraction causes a change in the direction of the x-ray path, which only can be measured indirectly using for example an interferometer as shown in Figure 1. This method provides the phase-contrast and the absorption-contrast images simultaneously. The device used with coherent synchrotron sources consists of two x-ray optical gratings [26], [27]: the phase grating produces an interference pattern in discrete distances downstream the x-ray beam, and the analyzer grating is used to detect the pattern of several micrometer periods with a standard x-ray imaging detector by scanning the analyzer grating with respect to the phase grating [21]. The method can be transferred to standard laboratory sources by adding an additional source grating, which increases the coherence of the X-ray source.

### Experimental setup

For the experimental setup a three grating interferometer has been used in combination with an ENRAF Nonius rotating anode X-ray tube and a single photon counting detector (Pilatus II, Dectris, Baden, Switzerland) with a pixel size of 172×172 µm^2^ and a silicon sensor thickness of 450 µm. Gratings with periods of 5.4 µm (Microworks, Karlsruhe, Germany) in a symmetric setup have been chosen: Both inter-grating distances have been set to 875 mm. The phase grating made of Nickel had a height of 8 µm and introduced a phase-shift of Π at the design energy of 22.8 keV. The golden source and analyzer grating structures were 50 µm high. The 6*^th^* Talbot order for the design energy of 23 keV was chosen and the mean visibility of the interferometer reached 13%. The X-ray filtration consisted of the following components:

- Beryllium window of the X-ray tube with a thickness of 0.25 mm
- Silicon wafers of source, phase and analyzer grating with a thickness 0.5 mm each
- Silicon disk with a thickness of 1 mm
- Approximately 2.25 m of air.

Beryllium window and silicon wafers of the gratings are not chosen based on advanced simulations but are parts of the individual components of the set-up. The silicon disk has been introduced to further reduce low energy photons without relevant reduction of photon flux.

The target material of the X-ray tube was Molybdenum and a tube voltage of 35 kVp has been applied in all tomography scans. The tube current has been set to 70 mA resulting in a total tube power of 2.45 kW. Altogether, without an object in the beam, an average count of 630 photons per second has been collected in each pixel of the detector. The actual size of the focal spot is 3×0.3 mm^2^, the effective source size is only 0.3×0.3 mm^2^ due to an inclination of the anode surface with respect to the optical axis of the beam.

The whole interferometer has been installed 60 cm away from the source, each sample was placed 70 mm in front of the phase grating and the detector 40 mm behind the analyzer grating, which gives a sample magnification of 1.72 and an effective pixel size of 0.1×0.1 mm^2^.

### Liver samples

All samples were acquired through the Institute of Pathology, Technische Universität München. They were initially resected for clinical reasons independently of this study. The patients provided their written consent to the use of tissue samples for educational and scientific purposes in general. The use of these specimens and the way how written consent was obtained has been fully approved by the ethics committee at the Faculty of Medicine of the Technische Universität München.

After excision a selected representative tissue sample was excised and put in a 50 ml plastic container in a 4% neutral-buffered formaldehyde solution. The following five different liver specimens with lesions have been chosen: 1.) a metastasis of a low-grade adenocarcinoma in a steatotic liver, 2.) a cholangiocellular carcinoma in a liver with macrosteatosis and pilosis, 3.) a hepatocellular carcinoma in a cirrhotic liver, 4.) a subcapsular hematoma and 5.) a metastasis of a mucinous adenocarcinoma of the colon. Histological sections of the specimens were prepared with a standard hematoxylin and eosion (H&E) staining. The slice thickness was 5 micrometers. Histology and PCCT slices were manually correlated using prominent features for the correct orientation.

### Image acquisition

The liver samples were put into cylindrical plastic containers of 30 mm diameter that were scanned in air. 1200 projections over 360° were recorded per tomography scan. Every 20 projections 5 reference projections without the sample were recorded for background correction. Each projection was obtained by the phase stepping method [19] using eleven images with 5 seconds exposure time acquired over one period of the source grating. The total scan time was approximately 25 h.

### Image reconstruction and post processing

**Figure 2:**
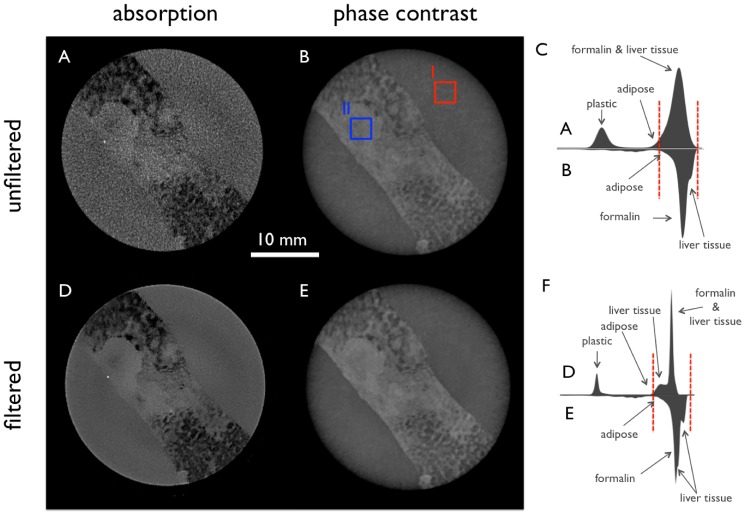
Comparison of unfiltered and filtered tomographic slices of a metastasis of a low-grade adenocarcinoma in a steatotic liver. Axial slices of the conventional absorption-based (A, D) and the phase-contrast (B, E) tomography windowed using the same window level and window width. The histograms of both unfiltered (C) and filtered (F) tomographic datasets demonstrate the filtering result and the red dashed markers show the window level. The areas I (red, surrounding formalin) and II (blue, high-contrast tumor in (B) mark the regions of 30×30 pixels, which were averaged for CNR calculation.

The beam divergence is approximately 1.1° and allows to perform a standard parallel beam filtered back-projection using a Ram-Lak filter to reconstruct the absorption-contrast data and a Hilbert filter to reconstruct the phase-contrast data [21]. The reconstructed slice thickness of 100 µm is given by the effective pixel size of the detector, and the vertical number of detector pixels limits the number of slices to 195. Joined bilateral filtering as described in [28] was used as post processing in order to reduce the noise level. This method exploits the fact that the absorption and phase-contrast images are perfectly registered with respect to each other by performing edge-preserving filtering where edges are detected simultaneously in both images. This method overcomes at least partially the problem of standard edge-preserving filtering where edges with a contrast-to-noise-ratio (CNR) less than 1 are typically not detected as edges and are thus smoothed. Due to the simultaneous detection of edges in both images, edge detection becomes more reliable and thus, a mutual benefit is created. For the liver samples here, the noise levels, which are free parameters in the joined bilateral filter, were estimated as the local standard deviation of reconstructed image values in homogeneous regions. The filter was applied using a 13^3^ neighborhood. The contrast-to-noise ratios (CNR) of two different regions (30×30 pixels each) of one sample relative to the surrounding formalin were calculated in absorption and phase-contrast tomogram before and after the denoising. The regions used for the CNR calculation are marked in Figure 2B The following equation was used to determine the CNRs:
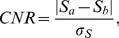
(1)where 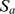 and 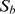 represent the averaged measured signals in the region 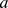 and 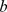, respectively, and 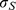 is the error of the averaged signal. Here we set 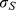 = 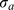, which is the standard deviation of the averaged signal 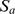 outside the specimen, to avoid higher noise values when averaging over inhomogeneous tissue. Please note that this implies the assumption that the noise is equally distributed over the whole image and the standard deviation 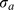 of the homogenous region outside the specimen represents the noise level in the image.

## Results

Figure 2 compares the unfiltered and the filtered tomographic slices of a metastasis of a low-grade adenocarcinoma in a steatotic liver. For this comparison the same window level and window width was used for the slices of both contrasts, which is indicated by red dashed lines in the histograms of the unfiltered (C) and of the filtered slices (F). The unfiltered absorption images (A) show a very low soft-tissue contrast and a high noise level. The areas with steatotic liver tissue show low density compared to the surrounding formalin, while the infiltrating tumor tissue in the center has a higher density. In the phase-contrast images (B, E) the steatotic liver tissue has also a low signal, but the tumor tissue shows intermediate to high signal with more details compared to the absorption images. Both signals profit from the post processing with the bilateral filter, but especially the absorption-contrast image contains many structural details inside the tumor after the filtering. For the remaining samples only the filtered images will be shown. To quantify the image-quality improvement the CNR of one region inside the tumour compared to the formalin was calculated for both signals. Note, that the two tomograms are intrinsically registered, thus, the marked regions-of-interest (ROIs) I (red, surrounding formalin) and II (blue, high-contrast tumor tissue) in Figure 2B correspond to 30×30 pixels and are exactly the same for the slices shown in Figures 2A, 2D, and 2E. These ROIs were averaged to determine the CNRs as described in the methods and materials section, where the signal 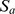 was chosen from the ROI I and the signal 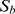 from the ROI II. The resulting CNRs are given in Table 1.

**Table 1:** Comparison of CNRs for unfiltered and filtered tomographic slices of a metastasis of a low-grade adenocarcinoma in a steatotic liver shown in Figure 2.

	absorption	absorption	phase-contrast	phase-contrast
	unfiltered	filtered	unfiltered	filtered
CNR between ROI I and II	0.39	1.24	5.34	8.63

For the calculation of the CNRs the areas I (red, surrounding formalin) and II (blue, high-contrast tumor) marked in Figure 2B were used, which correspond to 30×30 pixels.

**Figure 3:**
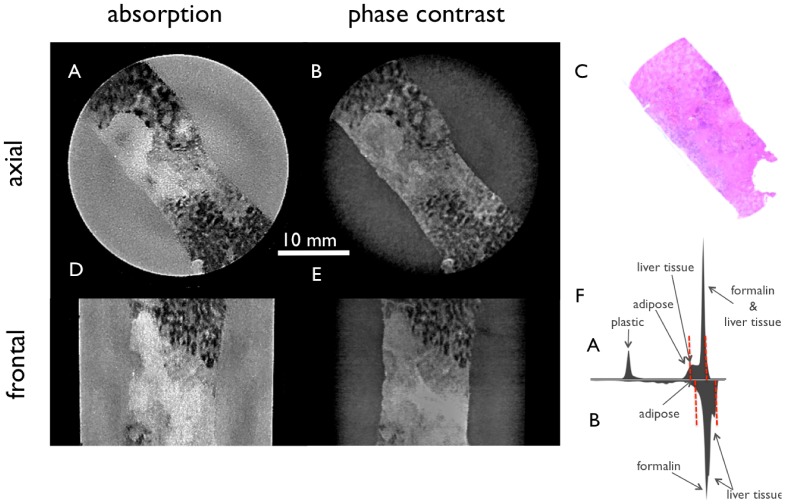
Filtered tomographic slices of a metastasis of a low-grade adenocarcinoma in a steatotic liver filtered with the 3D bilateral filter. Conventional absorption-based (A, D) and phase-contrast (B, E) CT images of the liver specimen in two different planes. H&E stain (C) corresponds to image A and B, respectively. (F) shows the histogram of the filtered absorption image (top) and the filtered phase-contrast image (bottom) with red dashed lines marking the window level in the shown slices.

In Figure 3 only the filtered tomographic slices of the same metastasis as shown in Figure 2 are depicted. The conventional absorption based (3A, 3D) and phase-contrast (3B, 3E) CT images of the liver specimen are shown in two different planes axial and frontal. The H&E stained histopathology image (3C) shows a corresponding region of the specimen like in 3A and 3B, respectively. The histograms (3F) of both filtered signals are presented to indicate the different windowing of the images, which is set to a visual optimum for each signal. The absorption images (3A, 3D) profit mostly from the post-processing in the higher-density area of the infiltrating tumor tissue in the center, while the areas with steatotic liver tissue with low density lead to an CNR improvement of this region in the phase-contrast image.

**Figure 4:**
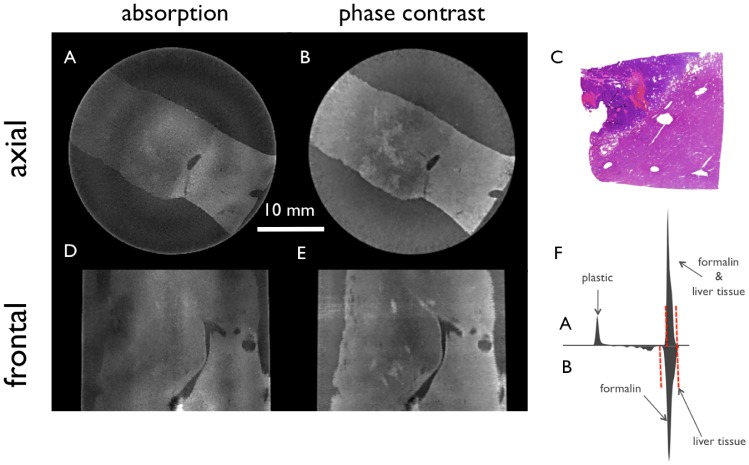
Tomographic slices of a cholangiocellular carcinoma in a liver with macrosteatosis and pilosis filtered with the 3D bilateral filter. Conventional absorption-based (A, D) and phase-contrast (B, E) CT images of the liver specimen in two different planes. H&E stain (C) corresponds to image A and B, respectively. (F) shows the histogram of the filtered absorption image (top) and the filtered phase-contrast image (bottom) with red dashed lines marking the window level in the shown slices. The absorption images (A, D) profit strongly from the filtering, but the information is still complementary.

Figure 4 shows filtered tomographic slices of a cholangiocellular carcinoma in a liver with macrosteatosis and pilosis. (4A, 4D) present the conventional absorption-based and (4B, 4E) the phase-contrast CT slices of the liver specimen in two different planes. In (4C) the H&E stained slice corresponding to the regions of images 4A and 4B is shown. Even after the bilateral filtering the soft-tissue contrast in absorption images (4A and 4D) is low in comparison with the phase-contrast images (4B, 4E), which show low signal for the tumor tissue in the center of the slice with high signal in necrotic/hemorrhagic areas compared to the normal liver tissue in the upper left and lower right parts (4B) with intermediate signal. Here, the windowing is chosen to a visual optimum of each signal and is indicated in the histograms (4F).

**Figure 5:**
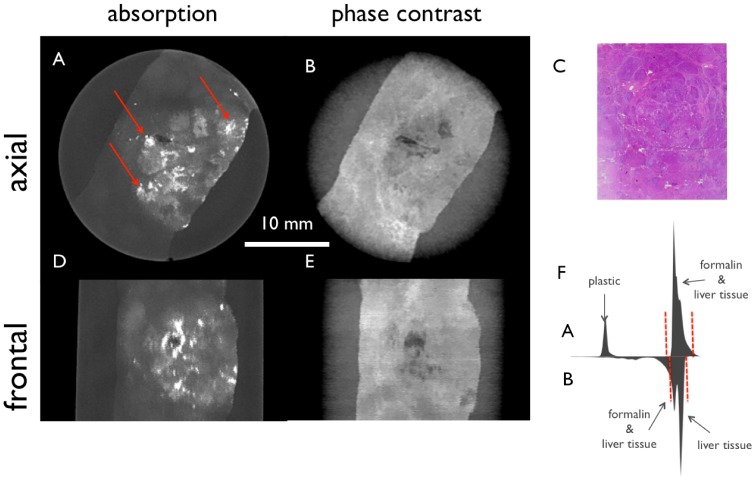
Filtered tomographic slices of a hepatocellular carcinoma in a cirrhotic liver. The patient had received transcatheter arterial chemoembolisation (TACE). Conventional absorption-based (A, D) and phase-contrast (B, E) CT images of the liver specimen in two different planes. H&E stain (C) corresponds to image A and B, respectively, and (F) shows the histogram of the absorption (top) and the phase-contrast image (bottom) with red dashed lines marking the window level. In the absorption images (A, D) the HCC nodules in the right center (marked with red arrows in A) are visible due to the high density of the retained Lipiodol. In the phase-contrast images (B, D) the fibrous septa are visible as high signal bands whereas the liver tissue and the HCC nodules show intermediate signal. Some areas with increased Lipiodol retention show low signal.

The filtered tomographic slices of a hepatocellular carcinoma in a cirrhotic liver are shown in Figure 5. The patient had received a transcatheter arterial chemoembolisation (TACE) prior to excision. In the absorption images (5A, 5D) the HCC nodules (marked with red arrows in 5A) in the right center are visible due to the high density of the retained Lipiodol. Lipiodol is a composition of iodine with poppyseed oil, nowadays mainly used for chemoembolization procedures where a chemotherapeutic agent is mixed with the oily liquid. Chemoembolization interrupts the tumors blood supply and allows high local concentrations of the drug for a longer period of time. In the phase-contrast images (5B, 5D) the fibrous septa are visible as high signal bands whereas the liver tissue and the HCC nodules show intermediate signal. Some areas with increased Lipiodol retention show low signal. Again the windowing is set to the visual optimum of each signal, which is marked in the histograms (5F).

**Figure 6:**
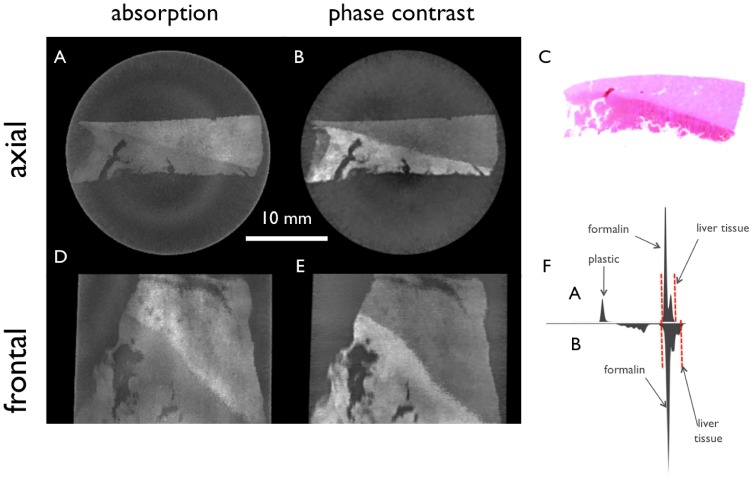
Filtered tomographic slices of a subcapsular hematoma. Conventional absorption- based (A, D) and phase-contrast (B, E) CT images of the liver specimen in two different planes. H&E stain (C) corresponds to image A and B, respectively, and the histogram (F) of the absorption (top) and the phase-contrast image (bottom) show the window level as red dashed lines. In the absorption images (A, D) the contrast between the liver tissue in the upper right part of the image (A, D) and the subcapsular hematoma in the lower left part is considerably lower compared to the phase-contrast image (B, E) where the hematoma shows a high signal.

Figure 6 presents the tomographic slices of a subcapsular hematoma after bilateral filtering. The contrast between the liver tissue in the upper right part of the images and the subcapsular hematoma in the lower left part is considerably lower in the absorption images (6A and 6D) compared to the phase-contrast images (6B and 6E), where the hematoma shows a high signal.

**Figure 7:**
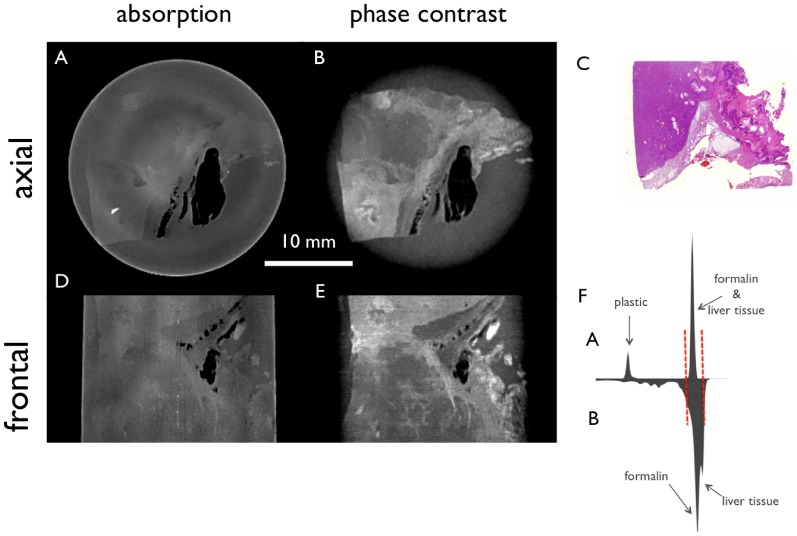
Filtered tomographic slices of a liver metastasis of a mucinous adenocarcinoma of the colon. Conventional absorption-based (A, D) and phase-contrast (B, E) CT images of the liver specimen in two different planes. H&E stain (C) corresponds to image A and B, respectively, and (F) shows the histograms of both signals with red dashed lines marking the window level. In the absorption images (A, C) only the mucinous parts of the metastasis are visible as areas with low density. The phase-contrast images (B, E) show a significantly higher soft-tissue contrast with low signal in the mucinous areas, intermediate signal in the liver tissue and higher signal for the tumor tissue and necrotic/hemorrhagic areas.

The last specimen was a metastasis of a mucinous adenocarcinoma of the colon. The corresponding filtered tomographic slices are shown in Figure 7. The phase-contrast images (7B, 7E) show a significantly higher soft-tissue contrast with low signal in the mucinous areas, intermediate signal in the liver tissue and higher signal for the tumor tissue and necrotic/hemorrhagic areas. In the absorption images (7A, 7C) only the mucinous parts of the metastasis are clearly visible as areas with low density. The other structures in the absorption images are barely visible since the contrast after filtering is in the same order of magnitude as the remaining low-frequency artifacts. Nevertheless, sharp edges are visible, which have been reliably detected in the phase-contrast image.

## Discussion

Our results clearly demonstrate that the tumor boundary and the soft-tissue in general can be visualized with a lab-based imaging setup. The effective pixel sizes of 100 µm are one magnitude larger than the previously reported experimental results on human liver tissue [29] and on other tissue types gained at brilliant synchrotron sources [25], [30]. This is not yet in the range of clinically relevant spatial resolution (i.e. 500 µm instead of 100 µm in our study) required to meet the stringent dose requirements for in-vivo applications [31], but it is the first experimental demonstration of the still present information gain when using PCCT in combination with polychromatic radiation and low spatial resolution for ex vivo human liver samples.

By the use of joined bilateral filtering, the CNR in both images was improved substantially. In the present study, most of the originally reconstructed phase-contrast images showed a much better CNR than the absorption-contrast images. Therefore, there was less mutual benefit but more a unidirectional one, viz. the absorption-contrast image gained much more than the phase-contrast image: Edges, which were completely invisible in the original FBP images were made visible thanks to their visibility in the phase-contrast image. However, we also found some high-contrast regions in the absorption-contrast image having a better CNR and in this region, the differential phase-contrast image profited from the joined processing. With regard to the different visible structures inside the liver specimens, especially the combination of both information has the potential to improve the diagnostics without any use of contrast agents in future.

The low-frequency noise (rings), which can be observed in the attenuation images, is a combination of a beam-hardening effect and remaining fringes in the background, which are not removed by the normalization with a flat-field image. We did not apply any corrections for these effects to our data.

Our system has some limitations, which need to be discussed in more detail. One of the limitations of our study concerns the formalin fixation of the specimens. Especially this fact might change the contrast for different tissue types compared to in-vivo imaging of the tissue surrounded by circulating blood. Further studies are required to investigate the influence of formalin fixation on the contrast compared to fresh tissue in both, absorption and phase-contrast data.

The second important limitation is related to the fact that our laboratory setup is still in an experimental state. This means that it was not optimized for fast measurements, which makes it comparable to MRI with a microscopy coil in terms of scanning times, but allowing better spatial resolution. Optimization of other parameters, which are important for the phase-contrast imaging (as for example filtering of the spectrum or setup geometry) are subject of ongoing studies, and are non-trivial in combination with a polychromatic source of a limited flux. These parameters strongly influence the quality of the interferometer and its sensitivity, but at the same time can reduce the number of photons, which downgrades the image quality. Thus, our measurements do also not meet any dose requirements for in-vivo applications.

The applied dose can be roughly estimated from the number of the photons recorded by the detector. Approximately 600 photons were collected per image, per second and detector pixel. Taking into account the absorption of photons by the analyzer grating (50%), the quantum efficiency of the Pilatus detector (8% @ 35 keV and 25% @ 22 keV) and the effective source spectrum, we can estimate that the total applied dose to the sample was in the order of several Gy for a full tomography scan. Due to a non-optimized setup only a small fraction of the total applied dose really contributed to the signal formation and detection. Nevertheless, the comparison of the presented images of both signals - absorption and phase-contrast - is fair, since both were obtained simultaneously and at exact the same dose.

Various optimization steps applied to our system can bring the applied dose per scan into the range of conventional micro CT systems (e.g. small-animal micro CT systems), which have comparably high spatial resolution and apply several hundreds of mGy for a full tomography scan.

Optimization can be done from the detector side improving the detection efficiency of X-rays in the used energy range. The relatively low detector efficiency of our experimental system results in low photon statistic, which especially becomes apparent in the absorption images, where the image quality is worse than expected from measurements with an applied dose of several Gy. Modern detectors used in clinical systems can reach a quantum efficiency of up to 60% [32], which brings us already into the ball park of several hundreds mGy used in small animal scanning systems. A doubling of the visibility allows a dose reduction to a quarter at constant image quality [33]. Thus, raising visibility from currently 10% to 30% by optimization of gratings and setup geometry would reduce dose to 1/9 of the current value.

Advanced acquisition protocols can also reduce the acquisition time and the applied dose, which has been successfully demonstrated for grating-based imaging either theoretically or experimentally at synchrotron sources (low-dose grating-based phase-contrast imaging [34] and advanced stepping methods [35]). We also anticipate further possibilities to maintain the current CNRs at reduced dose and thus at reduced scan time by using iterative reconstruction techniques. In our experimental study the acquisition parameters like number of projections, number of phase steps, and acquisition time were arbitrarily chosen. Optimizing these parameters can significantly reduce the dose, e.g. utilizing a single-shot technique will allow a reduction of dose and acquisition time by a factor of 11 (as we have used 11 steps in the phase-stepping scan in our study) [36]. Furthermore, recently published results demonstrate that novel reconstruction methods can reduce dose and scanning time in phase-contrast CT of breast tissue by up to 74% [37].

The field-of-view of the grating-based imaging method is currently limited by the size of the available grating structures. Different efforts were made to overcome this limitation, by e.g. bending the wafer of large-area grating structures to account for the cone beam used for large field-of-views [38]. Recently, we could demonstrate that the grating-based method is able to work at high X-ray energies (up to 82 keV [39]), which brings it already in the range of clinically relevant X-ray energies. Further investigations are required to demonstrate the ability of the imaging method to image large objects in the range of the human body (20–40 cm diameter), which might lead to a degradation of image quality due to the non-negligible amount of scattering by large objects.

In conclusion, with our work we demonstrate the feasibility to assess the potential of phase-contrast imaging for different kind of liver diseases on ex vivo samples using laboratory-based X-ray generators. Our method provides a three-dimensional map of the attenuation coefficients and the electron density distribution of the specimen at the same time and the joined filtering combines the information to improve the image quality.

## References

[1] Lanitis S, Zacharioudakis C, Zafeiriadou P, Armoutides V, Karaliotas C, et al (2012) Incidental findings in trauma patients during focused assessment with sonography for trauma. American Surgeon 78: 366–372. 22524779

[2] Seitz K, Greis C, Schuler A, Bernatik T, Blank W, et al (2011) Frequency of tumor entities among liver tumors of unclear etiology initially detected by sonography in the noncirrhotic or cirrhotic livers of 1349 patients. Results of the DEGUM multicenter study. Ultraschall in der Medizin (Stuttgart, Germany : 1980) 32: 598–603. 10.1055/s-0031-1281858 22161557

[3] Völk M, Strotzer M, Lenhart M, Techert J, Seitz J, et al (2001) Frequency of benign hepatic lesions incidentally detected with contrast-enhanced thin-section portal venous phase spiral CT. Acta radiologica (Stockholm, Sweden : 1987) 42: 172–5. 10.1034/j.1600-0455.2001.042002172.x 11259945

[4] Berland LL, Silverman SG, Gore RM, Mayo-Smith WW, Megibow AJ, et al (2010) Managing incidental findings on abdominal CT: white paper of the ACR incidental findings committee. Journal of the American College of Radiology : JACR 7: 754–73. 20889105 10.1016/j.jacr.2010.06.013

[5] Sahani DV, Kalva SP (2004) Imaging the liver. Nursing Standard Official Newspaper Of The Royal College Of Nursing 10: 55–56.

[6] Oudkerk M, Torres CG, Song B, König M, Grimm J, et al (2002) Characterization of liver lesions with mangafodipir trisodium-enhanced MR imaging: multicenter study comparing MR and dualphase spiral CT. Radiology 223: 517–524. 11997562 10.1148/radiol.2232010318

[7] Stacul F, van der Molen AJ, Reimer P, Webb JAW, Thomsen HS, et al (2011) Contrast induced nephropathy: updated ESUR Contrast Media Safety Committee guidelines. European radiology 21: 2527–41. 21866433 10.1007/s00330-011-2225-0

[8] Der Molen AJV, Thomsen HS, Morcos SK (2004) Effect of iodinated contrast media on thyroid function in adults. European Radiology 14: 902–907. 14997334 10.1007/s00330-004-2238-z

[9] Thomsen HS, Morcos SK, Almén T, Aspelin P, Bellin MF, et al (2010) Metformin and contrast media. Radiology 256: 672–3 author reply 673. 20656850 10.1148/radiol.100566

[10] Cochran ST, Bomyea K, Sayre JW (2001) Trends in adverse events after IV administration of contrast media. Ajr American Journal Of Roentgenology 176. 10.2214/ajr.176.6.1761385 11373197

[11] Eiber M, Fingerle AA, Brügel M, Gaa J, Rummeny EJ, et al (2012) Detection and classification of focal liver lesions in patients with colorectal cancer: retrospective comparison of diffusion-weighted MR imaging and multi-slice CT. European journal of radiology 81: 683–91. 21316886 10.1016/j.ejrad.2011.01.072

[12] Holzapfel K, Eiber MJ, Fingerle AA, Bruegel M, Rummeny EJ, et al (2012) Detection, classification, and characterization of focal liver lesions: Value of diffusion-weighted MR imaging, gadoxetic acid-enhanced MR imaging and the combination of both methods. Abdominal imaging 37: 74–82. 21597893 10.1007/s00261-011-9758-1

[13] Momose A (2005) Recent advances in X-ray phase imaging. Japanese Journal of Applied Physics 44: 6355–6367.

[14] Bonse U, Hart M (1965) An X-Ray Interferometer. Appl Phys Lett 6: 155–156.

[15] Beckmann F, Heise K, Kölsch B, Bonse U, Rajewsky MF, et al (1999) Three-Dimensional Imaging of Nerve Tissue by X-Ray Phase-Contrast Microtomography. Biophysical Journal 76: 98–102. 9876126 10.1016/S0006-3495(99)77181-X PMC1302503

[16] Cloetens P, Ludwig W, Baruchel J, van Dyck D, van Landuyt J, et al (1999) Holotomography: Quantitative phase tomography with micrometer resolution using hard synchrotron radiation x rays. Applied Physics Letters 75: 2912–2914.

[17] Davis TJ, Gureyev TE, Gao D, Stevenson AW, Wilkins SW (1995) X-ray Image Contrast from a Simple Phase Object. Physical Review Letters 74 16: 3173–3177. 10058130 10.1103/PhysRevLett.74.3173

[18] Dilmanian FA, Zhong Z, Ren B, Wu XY, Chapman LD, et al (2000) Computed tomography of x-ray index of refraction using the diffraction enhanced imaging method. Physics in Medicine and Biology 45: 933–946. 10795982 10.1088/0031-9155/45/4/309

[19] Weitkamp T, Diaz A, David C, Pfeiffer F, Stampanoni M, et al (2005) X-ray phase imaging with a grating interferometer. Optics Express 13: 6296–6304. 19498642 10.1364/opex.13.006296

[20] Momose A, Yashiro W, Takeda Y, Suzuki Y, Hattori T (2006) Phase Tomography by X-ray Talbot Interferometry for Biological Imaging. Japanese Journal of Applied Physics 45: 5254–5262.

[21] Pfeiffer F, Kottler C, Bunk O, David C (2007) Hard X-Ray Phase Tomography with Low-Brilliance Sources. Physical Review Letters 98: 108105. 17358572 10.1103/PhysRevLett.98.108105

[22] Bech M, Bunk O, David C, Ruth R, Rifkin J, et al (2009) Hard X-ray phase-contrast imaging with the Compact Light Source based on inverse Compton X-rays. Journal of Synchrotron Radiation 16: 43–47. 19096173 10.1107/S090904950803464X PMC2724997

[23] Donath T, Pfeiffer F, Bunk O, Gruenzweig C, Hempel E, et al (2010) Toward Clinical X-ray Phase-Contrast CT Demonstration of Enhanced Soft-Tissue Contrast in Human Specimen. Investigative Radiology 45: 445–452. 20498610 10.1097/RLI.0b013e3181e21866

[24] Jensen TH, Bech M, Bunk O, Donath T, David C, et al (2010) Directional x-ray dark-field imaging. Physics in Medicine and Biology 55: 3317–3323. 20484780 10.1088/0031-9155/55/12/004

[25] Schulz G, Weitkamp T, Zanette I, Pfeiffer F, Beckmann F, et al (2010) High-resolution tomographic imaging of a human cerebellum: comparison of absorption and grating-based phase contrast. Journal of The Royal Society Interface 10.1098/rsif.2010.0281 PMC2988270 20659930

[26] David C, Bruder J, Rohbeck T, Grünzweig C, Kottler C, et al (2007) Fabrication of diffraction gratings for hard X-ray phase contrast imaging. Microelectronic Engineering 84: 1172–1177.

[27] Reznikova E, Mohr J, Boerner M, Nazmov V, Jakobs PJ (2008) Soft X-ray lithography of high aspect ratio SU8 submicron structures. Microsystem Technologies 14: 1683–1688.

[28] Koehler T, Roessl E (2012) Simultaneous de-noising in phase contrast tomography. AIP Conference Proceedings 1466: 78–83.

[29] Noël PB, Herzen J, Fingerle AA, Willner M, Stockmar MK, et al (2013) (Not yet published) Evaluation of the potential of phase-contrast computed tomography for improved visualization of cancerous human liver tissue. Zeitschrift fur medizinische Physik 10.1016/j.zemedi.2013.02.006 23570951

[30] Sztrókay A, Herzen J, Auweter S, Liebhardt S, Mayr D, et al (2012) Assessment of grating-based X-ray phase-contrast CT for differentiation of invasive ductal carcinoma and ductal carcinoma in situ in an experimental ex vivo set-up. European Radiology 1–7. 10.1007/s00330-012-2592-1 22932738

[31] Raupach R, Flohr T (2012) Performance evaluation of x-ray differential phase contrast computed tomography (PCT) with respect to medical imaging. Medical Physics 39: 4761–4774. 22894401 10.1118/1.4736529

[32] Ghetti C, Borrini A, Ortenzia O, Rossi R, Ordóñez PL (2008) Physical characteristics of GE Senographe Essential and DS digital mammography detectors. Medical physics 35: 456–63. 18383665 10.1118/1.2828185

[33] Köhler T, Jürgen Engel K, Roessl E (2011) Noise properties of grating-based x-ray phase contrast computed tomography. Medical physics 38 Suppl 1: S106. 21978111 10.1118/1.3532396

[34] Zanette I, Bech M, Rack A, Le Duc G, Tafforeau P, et al (2012) Trimodal low-dose X-ray tomography. Proceedings of the National Academy of Sciences 109: 10199–10204. 10.1073/pnas.1117861109 PMC3387041 22699500

[35] Zanette I, Bech M, Pfeiffer F, Weitkamp T (2011) Interlaced phase stepping in phase-contrast x-ray tomography. Applied Physics Letters 98: 94101.

[36] Bevins N, Zambelli J, Li K, Qi Z, Chen GH (2012) Multicontrast x-ray computed tomography imaging using Talbot-Lau interferometry without phase stepping. Medical physics 39: 424–8. 22225312 10.1118/1.3672163 PMC3261056

[37] Zhao Y, Brun E, Coan P, Huang Z, Sztrókay A, et al (2012) High-resolution, low-dose phase contrast X-ray tomography for 3D diagnosis of human breast cancers. Proceedings of the National Academy of Sciences of the United States of America 109: 18290–4. 23091003 10.1073/pnas.1204460109 PMC3494902

[38] Danielsson M, Cederwall B, Carlson P, Bohm C, Revol V, et al (2011) X-ray interferometer with bent gratings: Towards larger fields of view. Nuclear Instruments and Methods in Physics Research Section A: Accelerators, Spectrometers, Detectors and Associated Equipment 648: S302–S305.

[39] Willner M, Bech M, Herzen J, Zanette I, Hahn D, et al (2013) Quantitative X-ray phase-contrast computed tomography at 82 keV. Opt Express 21: 4155–4166. 23481949 10.1364/OE.21.004155

